# Behind Enemy Lines: Immunomodulatory Armamentarium of the Schistosome Parasite

**DOI:** 10.3389/fimmu.2020.01018

**Published:** 2020-06-09

**Authors:** Jose Ma. M. Angeles, Van Jerwin P. Mercado, Pilarita T. Rivera

**Affiliations:** Department of Parasitology, College of Public Health, University of the Philippines Manila, Manila, Philippines

**Keywords:** schistosomiasis, immunomodulation, innate immunity, parasite, Th2 immune response, cytokines

## Abstract

The deeply rooted, intricate relationship between the *Schistosoma* parasite and the human host has enabled the parasite to successfully survive within the host and surreptitiously evade the host's immune attacks. The parasite has developed a variety of strategies in its immunomodulatory armamentarium to promote infection without getting harmed or killed in the battlefield of immune responses. These include the production of immunomodulatory molecules, alteration of membranes, and the promotion of granuloma formation. Schistosomiasis thus serves as a paradigm for understanding the Th2 immune responses seen in various helminthiases. This review therefore aims to summarize the immunomodulatory mechanisms of the schistosome parasites to survive inside the host. Understanding these immunomodulatory strategies not only provides information on parasite-host interactions, but also forms the basis in the development of novel drugs and vaccines against the schistosome infection, as well as various types of autoimmune and inflammatory conditions.

## Introduction

Immunomodulation is a tactic employed by parasites to successfully invade their human hosts. As an adaptive survival skill, helminths employ a great diversity of immunomodulatory strategies for evading immune detection, suppressing cellular immunity, and eluding host immune attacks (1, 2). These promote their longevity inside the host to further continue their life cycle and facilitate transmission.

Unlike the rapidly multiplying protozoan parasites, some of which use antigenic variation as an effective evasion strategy in escaping immune recognition (3), the chronicity of helminthic infections has led helminths to act against the host immune responses by downmodulating the latter's intensity and effectiveness. *Schistosoma* parasites have been shown to induce the Th2 response that is shown to be more favorable to important biological processes inside the host such as migration and egg excretion (4). During its intra-mammalian life cycle, *Schistosoma* needs to conquer a war zone consisting of the host's innate and adaptive immune responses. The life stages of the parasite that will have to contend with the host's immune system are the penetrating cercariae, the migrating schistosomula, the adult worms, and the eggs produced by the adults *in copula*.

During schistosome infection in the mammalian host, cytokines play major roles in the regulation of immune and inflammatory responses against invading parasites. These effector molecules, particularly those produced by the immune cells, not only mediate both physiological and pathological consequences at the onset of immune response, but also control the degree and duration of such a response. The schistosome parasites are therefore equipped with immunomodulatory armamentarium acting as counter-defenses to protect themselves from the destruction brought about by host immune attacks. This review aims to provide a summary of these immunomodulatory strategies that might be crucial for the survival of the schistosomes within their definitive host.

## Infiltrating Host Territory

The skin is the largest organ of the body and consists of a complex network of different cell types that maintain several vital processes including immune responses for disease prevention. The schistosome cercariae begin their invasion by infiltrating the skin- the host's primary defense. During invasion, the parasite needs to ensure its survival by orchestrating immune regulation within the skin. The percutaneous entry of the schistosomula elicits an inflammatory response characterized by infiltration of polymorphonuclear cells (PMNs) and mononuclear cells (5, 6). Localized production of pro-inflammatory cytokines including interleukin (IL)-1b, IL-6, IL-12, and tumor necrosis factor (TNF)-α (7, 8) is supposed to promote this pro-inflammatory Th1 response. However, invasion by the schistosome paradoxically leads to predominantly Th2 immunity. This skewing of the immune response arises from the production of certain immunoregulatory mediators. IL-10, produced by keratinocytes, macrophages, dendritic cells (DCs) and B1 lymphocytes, is one of these key immunomodulatory cytokines elaborated in the skin in response to the cercarial invasion (9). In addition, a major molecule from the secreted protein of the cercaria known as Sm16 has been shown to modulate innate immunity by preventing macrophage classical activation and delaying antigen processing (10). Sm16 is also capable of blocking the activation of IL-1 receptor-associated kinase 1 (IRAK-1) gene, which is important in the production of nuclear factor kappa-light-chain enhancer of activated B cells (NF-κB) (11). NF-κB is known to be a key player in the regulation of immune responses to infection (12).

Excretory-secretory (ES) products from the cercaria likewise stimulate inhibitory molecules like prostaglandins. Studies have shown that schistosomula can induce prostaglandin E2 (PGE2) production in the human keratinocytes (13). This over-expression of PGE2 in the skin plays an important role in the production of IL-10 through a cyclooxygenase 2-dependent pathway (14). In addition, PGE2 is also a potent vasodilator (15) aiding the passage of the schistosome into the circulation. In fact, in a murine model for *Schistosoma mansoni*, PGE2 has been documented to be the main immunoregulatory molecule in the skin (13).

Langerhans cells (LCs) are considered the first-line fighters in the skin considering their location in the outer layers as compared with other types of DCs (16). LCs are known to induce immunological tolerance (17, 18), and their suppressive effects arise from IL-10 production and CD4+ regulatory T cells induction (19). When the skin is invaded by pathogens such as schistosomes, keratinocytes and LCs produce pro-inflammatory cytokines such as TNF-α and IL-1b stimulating the actin-dependent migration of the LCs (20). Another prostaglandin, now produced as a component of the schistosomula's ES proteins, prostaglandin D2 (PGD2) together with PGE2 leads to increased production of IL10 (21). The anti-inflammatory IL-10 downregulates the production of both IL-1b and TNF-α, thus inhibiting the migration of epidermal LCs to the site of invasion (21). Overall, this disruption in the movement of antigen presenting cells (APCs) from the site of exposure to the draining lymphoid tissue is a vital immunomodulatory strategy adopted by the *Schistosoma* parasites (21).

Mast cells (MCs) are another key player in the immune response against parasitic infections. MCs are abundant near cutaneous and mucosal body surfaces where early immune surveillance occurs. The schistosome parasite has been shown to release ES proteins that can induce mast cell degranulation (22). One of these molecules is likely to be the schistosome homolog of the human translationally controlled tumor protein (TCTP) that has been shown to degranulate both basophils and mast cells (23). Binding of histamine from activated mast cells to H2 histamine receptors induces IL-10 production in maturing DCs (24, 25) and inhibits the production of Th1 promoting cytokine IL-12, which in turn is a powerful inducer of interferon- γ (IFN-γ) (26). This results in matured DCs polarizing naive CD4+ T cells toward the Th2 phenotype (24).

Parasites can also regulate the host's immune response by inducing apoptosis of host cells (27). A 23 kDa protein called *S. mansoni* apoptosis factor (SMAF) has been characterized as a component of the cercarial ES products that can trigger apoptosis in the CD4+ lymphocyte population via Fas–FasL interaction. The same study suggests that the CD4+ cell apoptosis modulates the host's immune response and allows the schistosome parasite to evade immune surveillance (28).

Studies have also shown that ES products from the schistosomula stimulate APCs toward Th2 immune responses. ES-activated DCs trigger CD4+ cells to produce regulatory cytokines IL-4, IL-5, and IL-10-, all indicative of a Th2 response (29). Furthermore, these DCs also lose the ability to produce Th1-promoting cytokines including IL-12, IL-23, and IL-27 (30). It thus appears that immunomodulatory molecules in the ES products could modify the APCs to promote Th2 responses over the Th1 phenotype (31).

## Camouflaging of The Migrating Schistosomula

Skin-stage schistosomules are susceptible to both humoral and cellular immune responses. However, the significant morphological and biochemical changes occurring in the developing schistosomula render them resistant to the host immunological defenses (32), as seen in the lung schistosomulum (33, 34). These changes include shedding of the cercarial membrane and formation of the heptalaminate surface membrane (35). This unique outer-surface tegumental membrane might be an adaptation to resist host immune effectors such as complement activation and antibody-dependent cell-mediated cytotoxicity (ADCC) (36, 37). Different immune evasion strategies have been proposed to explain the inefficient host immune response against the exposed schistosome tegument (37). These include rapid tegument turnover, masking with acquired host antigens, and poor immunogenicity of exposed antigens (38).

Danger-associated molecular patterns (DAMPs) are tissue-derived distress signals released during stress or injury. One such DAMP is extracellular ATP involved in purinergic signaling (39). Extracellular ATP has been shown to induce the degranulation of neutrophils and the production of pro-inflammatory cytokines in macrophages and monocytes (40). Ecto-enzymes, such as alkaline phosphatase, phosphodiesterase, and ATP diphosphohydrolase, have been found to be expressed in the tegument of schistosomula (41–43). These ecto-enzymes might catalyze the conversion of ATP to adenosine and effectively degrade DAMPs released by host cells in response to intravascular schistosome migration, interfere with purinergic signaling, thus preventing pro-inflammatory responses, and subsequently lowering host immunity against the parasite (44).

Aside from their role against the pro-inflammatory ATP, these ecto-enzymes also inhibit blood coagulation in the tissue vicinity (45). Platelets themselves have been shown to damage the schisosome parasite (46). The catabolism of ATP and ADP through the ecto-enzymes characterized in *S. mansoni*, including the tegumental ecto-apyrase ATP diphosphohydrolase (SmATPDase-1) (47), alkaline phosphatase (SmAP) (48), and phosphodiesterase (SmNPP5) (49), may lead to the inhibition of platelet aggregation and thrombus formation around the worm. Moreover, activated platelets and immune cells release inorganic polyphosphates (polyPs) (50, 51). polyPs are essential for the activation of factor XII, which triggers the kallikrein-mediated kininogen pathway, thus producing high levels of bradykinin, increasing vascular permeability, and promoting inflammatory responses (52). SmAP has been shown to hydrolyze polyPs *in vitro* thereby possibly preventing their action against the parasite (48). This SmAP-mediated cleavage of polyPs may therefore contribute to the survival of the intravascular stages of the schistosome parasite, including the schistosomula and the adult pairs within their hostile habitat (48).

Lung schistosomula need to resist immune damage as they have been shown to activate complement (53) and bind antibodies on their surface membrane (54). Therefore, the structural and biochemical modifications of the schistosomulum's surface membrane tend to produce immunological camouflage that either prevent antibody binding or effectively reduce antigen expression (32). Furthermore, caveolin-like molecules and membrane fractions characteristic of detergent-insoluble glycosphingolipid-enriched membrane domains (DIGs) or detergent-resistant membranes (DRMs) have been observed on the surface membrane of the schistosome (55), thus indicating the presence of lipid-rafts (56) that might serve as an additional protection for the parasite. Lipid rafts are presumed to enable signal transduction by selectively concentrating intracellular signaling molecules in which protein kinases, scaffolding molecules, and substrates are in close proximity (57). In schistosomes, the lipid rafts have been shown to possess specialized signaling domains such as protein kinase C (PKC) and extracellular signal-regulated kinase (ERK) (58). PKC and ERK are important mediators known to regulate diverse processes in eukaryotes such as growth, development and differentiation, cell cycle, motility, apoptosis, and survival (59, 60).

Moreover, genes associated with immune evasion and stress responses, such as the potent anti-inflammatory Sm-16 and paramyosin, are over-expressed in lung schistosomula (61). Sm-16 might play a crucial role in the interaction of the parasite with immunoreactive lung microvasculature endothelial cells during the passage of the schistosomulum through the lung (62). On the other hand, paramyosin, found on the tegumental surface of the schistosomula, aids in immune evasion through its receptor that is capable of adsorbing antibodies onto the parasite surface at the latter's Fc regions (62, 63).

Nitric oxide (NO) plays a very important role both in the mammalian hosts and in helminths with respiratory pathology (64). It is a key messenger in the pathogenesis of inflammation by acting as a signaling molecule during T cell-mediated immunity (65). IFN-γ up-regulates inducible nitric oxide synthase (iNOS) leading to the production of NO (66). This cytokine is produced by the immune effector CD4+ T cells as an immune response against the schistosomula in the lungs (66). In an experimental study comparing the susceptibilities of different stages of larvae to killing by NO, lung schistosomula obtained 1 week after infection were not killed *in vitro* by NO generated either from a chemical NO donor or from activated cells (67). At this period, the schistosomula has been shown to undergo anaerobic metabolism (68), thus negating the aerobic metabolism-dependent effects of NO against the parasite (67). During transformation of cercaria into schistosomulum, the parasite rapidly shifts from carbon dioxide production via the Krebs cycle to lactate production using glycolysis (69), and from consumption of stored glycogen to dependence on host glucose as fuel (70). Furthermore, schistosomula have higher levels of mRNAs associated with anaerobic glucose metabolism (70) and lower expression of respiratory enzymes (71). As the schistosomes develop into adults, however, they regain a significant capacity to produce energy via aerobic metabolism (70).

Once the schistosomulum becomes successful in evading the host's immune response, it goes into the portal veins and matures into an adult over a period of 1–3 weeks. The male and female adult schistosomes pair up, adhere to the veins, bring forth 300–3,000 eggs, and escape host immunity for many years.

## Survival of The Adult Pairs in The Vascular System

The major task of the adult schistosomes is to produce eggs while surviving within the vascular system of the host. The circulatory system is home to various immune defenses including immune cells, phagocytes, complement proteins, and antibodies. However, the adult schistosomes are capable of avoiding the immune recognition system by coating their outer tegument with antigens from the hosts. Several studies have shown that the adult *Schistosoma* parasites were covered with immunoglobulins, β2 microglobulin, complement components, and other host antigens (72–75).

The complement system is an essential component of innate host immunity, and therefore schistosomes should protect themselves from complement-dependent cytotoxicity. To avoid complement-mediated auto-hemolysis, host erythrocytes are provided with a 70-kDa glycosylphosphatidylinositol (GPI)-anchored protein known as decay accelerating factor (DAF), which inhibits C3 convertases in both the classical and alternative pathways of the complement system (76). An *in vitro* experiment showed that adult schistosomes were capable of abstracting DAF or CD55 from host erythrocytes, which then serves as a valuable defense against the action of the complement system (77). The adult schistosomes are also provided with inhibitors of human complement activation on their tegument such as the trispanning orphan receptor of *S. haematobium* (Sh-TOR), a receptor that can bind specifically to human complement C2 (78). Finally, paramyosin is a known inhibitor of the complement membrane attack complex. It has been discovered that earlier known complement inhibitor SCIP-1 (34) is just a surface-exposed form of paramyosin (79). Paramyosin might therefore have some significance in the immunomodulation by inhibiting the activation of the terminal pathway of the complement system (79).

## Skewing of Immune Response

Following schistosomule migration, a Th1 immune response is elicited as characterized by a marked increase in IL-1 and IFN-γ induced by the worm antigens (80). The Th1 response persists for approximately 5 weeks. However, as the parasites mature, the immune response is skewed into the Th2 type (30). Experimental single-sex infections in mice models have shown that both male and female worms individually induce IL-4 production by CD4^+^ T cells and promote a Th2 response even before eggs are laid (31).

Toll-like receptors (TLRs) are a family of pattern recognition receptors expressed in cells of the innate immune system such as macrophages and DCs (81). Activation of TLRs induces Th1 immune response with a predominant production of IFN-γ by the CD4+T cells (82), in addition to Th1 promoting cytokines IL-12, IL23, and IL27 secreted by APCs (83). In *S. mansoni*, the TLR2 and TLR4 of DCs have been shown to recognize the schistosome specific phosphatidyl serine-containing lipid antigen lysophosphatidylserine (lyso-PS) (84), and lacto-N-fucopentose III (LNFPIII), respectively, in the worm's ES (85). These TLR-mediated signaling reduces the ability of the DCs to produce IL-12 and promotes a polarization toward a Th2 immune response instead of the Th1 type. Ligation of LNFPIII and the TLR4 in DCs by *Schistosoma* induces phosphorylation of mitogen-activated MAP kinase (MAPK) ERK (85). On the other hand, the schistosomal lyso-PS has been shown to induce activation of DCs promoting Th2 and regulatory T cell development via a TLR2-dependent mechanism (84). TLR2 ligation stabilizes the MAPK ERK, and stimulates the transcription factor c-Fos, thereby suppressing IL-12 production, and promoting polarization toward Th2 immune responses (86).

At 5–6 weeks post-infection, the adult female schistosomes start to release eggs after pairing with the male worm. The schistosome eggs evoke a host immunity that is more robust compared with the ineffective response mounted against invading cercariae and adult worms (87). Eggs of *S. haematobium* have been shown to elicit an immediate, initial response within 24 h upon release, marked by the induction of pro-inflammatory mediators such as TNF-α, on one end, and that of anti-inflammatory cytokines that include CCL11 (88). Schistosome eggs, viable or dead, are remarkably capable of inducing Th2 responses (89). This Th2 phenotype is characterized by the proliferation of Th2 cells, eosinophils, and basophils; elevated production of immunoregulatory cytokines IL-4, IL-5, and IL-13; and polarization of antibodies toward the IgG1 and IgE isotypes, and of macrophages toward M2 phenotype (90, 91). The ES proteins, such as the dimeric glycoprotein alpha-1 (α1) or IL-4 inducing principle of schistosome eggs (IPSE), and the hepatotoxic egg glycoprotein omega-1 (ω1), thus play important roles in the immunomodulation of the CD4+ effector responses (92–95). Specifically, the ribonuclease activity of ω1 protein in the ES of *S. mansoni* eggs is found to be essential in inducing Th2-type response in DCs (94).

## Journey of The Schistosome Eggs

Schistosome eggs exit the host either by traversing the intestinal wall into the intestinal lumen via mesenteric vessels for *S. mansoni* and *S. japonicum*, or through migration into the vesical lumen of the bladder for *S. haematobium* (96). This egg expulsion however is mostly host-dependent as the schistosome eggs lack any motility mechanisms (97). As egg passage into the intestine is not guaranteed, about half of all the deposited eggs accidentally go to the liver (98). In order to continue transmission, the schistosome parasites employ strategies to ensure successful egg transit into the environment (98–101). Extravasation in the blood vessels is promoted by angiogenesis, endothelial activation, and fibrinolytic activity induced by schistosome eggs. The eggshell contains the enzymes enolase and glyceraldehyde-3-P-dehydrogenase (102) that act as surface binding receptors to plasminogen (103, 104). It was proposed that once it has reached the intestine, the schistosome induces granuloma formation to promote egg excretion, while at the same time preventing severe immunopathology that may otherwise affect egg release (105). It was previously noted that schistosome egg excretion is an exquisite, immune-dependent process (106).

The polarization of Th cells determines the macrophage phenotype and granuloma formation. M2 macrophage phenotype or alternatively activated macrophages are needed in effective granuloma formation and confer protection against excessive damage of the eggs during their movement across the intestinal tissue (105). M2 phenotype is promoted by IL-4/IL-13 release from Th2 cells in *S. mansoni* infection (106). This has been proven by the impaired granuloma formation during schistosome infection in T cell derived IL-4 and IL-13 deficient mice inhibiting the egg release into the intestinal lumen (107, 108). These mechanisms may suggest that Th2 immune responses collaborate with egg-derived proteases in promoting egg release from intestinal tissues.

## Immunomodulation in The Granuloma Formation

Unlike in intestinal granulomata, where schistosome eggs have the ability to exit into the gut lumen, the eggs in the hepatic granuloma remain trapped, with the granuloma becoming fibrotic over time. Secretions from the trapped eggs are known to stimulate the CD4+ T cells initially to release Th1-type cytokines IL-2 and IFN-γ facilitating delayed-type hypersensitivity reaction and early granuloma formation (90). This immune response gradually shifts to the Th2 phenotype with the production of IL-4, IL-5, IL-10, and IL-13 (90, 109–113). It has been shown that hepatosplenic schistosomiasis, a severe form of the disease, is associated with increased levels of Th1 cytokines TNF-α and IFN-γ, and decreased levels of Th2 cytokine IL-5 in a study done using peripheral blood mononuclear cells from patients (114). This proves that the outcome of the disease is dependent on the type of immune response elicited by the parasite within the host. In addition, *S. mansoni* eggs were shown to secrete chemokine binding protein (smCKBP) that is believed to block certain chemokines from inducing granuloma formation while preferentially altering the cellular features of the granuloma (115). Both *in vitro* and *in vivo* experiments have demonstrated that smCKBP tends to prevent the interaction of chemokines such as CXCL8 with specific cellular receptors, as well as the activation and migration of immune cells such as neutrophils (115).

Granuloma formed during prolonged Th1 response and a dampened Th2 response have been shown to display decreased size and fibrosis owing to downregulation of inflammation and of collagen deposition (116). This phenomenon might be attributed to the dominance of neutrophils infiltrating the lesion during the initiation of granuloma formation (117). Neutrophils are recruited by egg-specific proteins (118) to the core of the granuloma leading to a neutrophil-mediated inflammatory response that causes tissue damage (117). In addition, intact live eggs and soluble egg antigen (SEA) can trigger the release of neutrophil extracellular traps (NETs) within the core of the granuloma, potentially limiting the pathogenic effects of parasite eggs (119). NETs are web-like structures consisting of de-condensed chromatin and histones produced by activated neutrophils and are thought to be involved in pathogen trapping, including parasites such as *Plasmodium falciparum* (120) and *Strongyloides stercoralis* (121). A previous study has shown that eggs trapped within the mesh of NETs remain viable and were not killed, as opposed to the effect of NETs as seen in other pathogens (119). This might suggest that NETs only serve to immobilize or restrict the movement of the schistosome eggs, without adversely affecting their viability.

At a later stage of the disease, neutrophils secrete granule proteins that can degrade collagen, the major component of fibrotic granulomas, thus limiting the size of the granulomas. *S. japonicum* granuloma has neutrophils that accumulate within the core as early as 8 days post-deposition (122, 123), and at the periphery as granuloma matures (124). This implies that neutrophils have different roles in the granuloma formation depending on the time of their recruitment and their location within the lesion.

Although CD4+ T cells generally dictate the granulomatous response to the eggs, other immune cells like CD8+ T cells, B cells, M2 macrophages and eosinophils are also as important in the regulation of granuloma formation (125–128). Eosinophil infiltration in the granuloma is mediated by IL-5 and IL-13 (112, 127, 129, 130). This Th2-driven eosinophil infiltration in the granuloma stands in contrast with the early Th1 granuloma, which is dominated by neutrophils. Aside from the destructive actions directed against miracidia within trapped eggs upon their degranulation (131), eosinophils are also responsible for the polarization of the immune response to Th2 type by producing IL-4 and IL-5 (132). Granuloma in *S. mansoni* has been noted to have more eosinophils than neutrophils, which is in contrast to the neutrophil dominated granuloma seen in *S. japonicum* (133). The number of eosinophils in *S. mansoni* infection were 60.60 ± 0.47%, and 44.30 ± 0.23% of all the granuloma cells in the acute and chronic experimental infections, respectively, using murine models for both hepatic and intestinal infection (134). In an earlier experiment done using murine models for lung granuloma, results showed about 70% of the cellular population in *S. mansoni* granuloma are eosinophils at 16 days post-deposition (125).

CD4+ T cells are the primary source of IL-13 (135), the dominant Th2 cytokine responsible for the development of liver fibrosis (136). Together with IL-4, IL-13 induces macrophage expression of arginase, which then cleaves L-arginine to form L-ornithine (136). Ornithine aminotransferase then converts L-ornithine to proline, which is important in collagen production and fibrosis development (137). IL-13 also triggers the trans-differentiation of hepatic stellate cells (HSCs), one of the main sources of hepatic collagen, and plays an important role in schistosome-induced fibrogenesis (138).

The granuloma both functions as a major pathology in schistosomiasis disease and as a protective barrier between the egg and the liver tissues. Although the Th1-dominated immune response gives rise to granulomas with smaller sizes and less fibrosis, the switch to the Th2 phenotype confers some protective effects to the host (139). The granuloma functions to sequester egg secretions that can otherwise cause damage to liver tissue (139).

## The Dual Role of TGF- β

Th17 serves as a unique CD4+ T cell subset and is characterized by IL-17 production as an adaptive host mechanism in cases where both Th1 and Th2 immune responses are inappropriate for protection against the pathogen (140). IL-17 is a pro-inflammatory cytokine often seen in the pathogenesis of autoimmune diseases. In *S. japonicum*, SEAs are believed to induce a Th17 response (141) linking it to severe hepatic inflammation in schistosomiasis (142, 143). The association between Th17 and the severity of the disease is also seen in *S. mansoni* infection as an exacerbation of granuloma in mice models is primarily directed by a Th17 response (144, 145). The role of IL-17 in granuloma formation is further proven by a decrease in the size of granulomata when anti-IL-17 neutralizing antibodies were given to infected mice (142).

Th17 differentiation is induced in mice exposed to transforming growth factor-β (TGF-β) and IL-6 (146–148). As TGF-β is also known to induce differentiation of CD4+ T cells into forkhead box protein 3 (FoxP3)-expressing regulatory T cells (Tregs) (149), the pivotal role of TGF-β in the progress of the disease makes it one of the most important cytokines that determines disease outcome. Th17 cells promote inflammation through the production of IL-17, IL-22, and IL-23, and neutrophil recruitment (150), whereas Tregs produce the anti-inflammatory cytokines IL-10 and TGF-β (151). Surprisingly, Tregs can be transformed into Th17 cells in the presence of IL-6 (152). Interestingly, Th17 cells appear to be resistant to Tregs' suppressive effects (153, 154). Thus, the delicate balance maintained between anti-inflammatory Tregs and pro-inflammatory Th17 cells is a prime determinant in disease severity. The imbalance of Th17/Treg has been shown to be closely associated with immunopathological damage and egg granuloma formation in mouse models infected with *S. japonicum* (155).

Currently, not much is known about how the balance between the Th17/Treg immune responses modulates disease progression in schistosomiasis. It is therefore worthwhile to elucidate which mechanisms promote Treg proliferation during the chronic phase of schistosome infection when Th2 immune responses start to wane and lead to immune hypo-responsiveness.

## Conclusion and Future Directions

**Figure 1:**
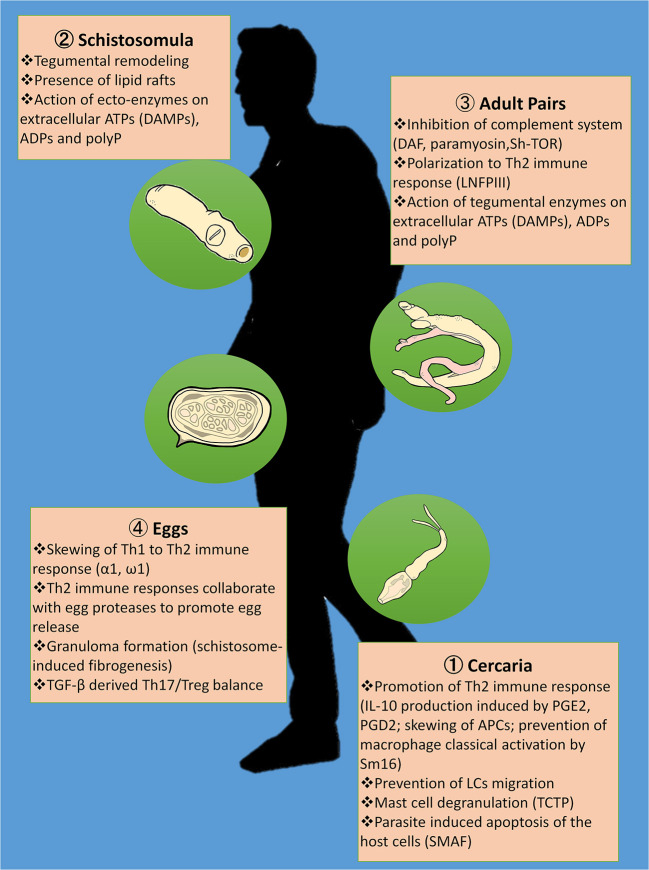
Summary of proposed immunomodulatory strategies of the schistosome in evading the host immune responses. Skewing of Th1 to Th2 immune response is very much evident during cercarial penetration **** through IL-10 production induced by prostaglandin E2 (PGE2) and prostaglandin D2 (PGD2), prevention of macrophage classical activation by Sm16 and disorientation of the antigen presenting cells (APCs); lodging of the adult schistosome pairs in the veins **** through the interactions between lacto-N-fucopentose III (LNFPIII) and toll like receptors (TLRs); and egg deposition in the intestinal and liver tissues **** through the immunomodulatory effects of the egg's excretory-secretory proteins (ES) to CD4+ effector responses including α1 and ω1. In addition, the cercariae **** are able to evade innate immunity in the skin by preventing migration of Langerhans cells (LCs), mast cell degranulation promoted by the translationally-controlled tumor protein (TCTP) homolog in schistosomes, and *Schistosoma mansoni* apoptosis-inducing factor (SMAF)-mediated host cell death. Schistosomula's **** tegumental remodeling and the presence of lipid rafts covering the parasite render them undetectable to immune responses during migration. Once the adult schistosomes **** settle in the mesenteric veins, they become capable of evading the host complement system through the abstraction of erythrocytes' decay-accelerating factor (DAF) as seen in *in vitro* studies, the binding of human complement C8 and C9 to the schistosome's paramyosin, and the attachment of C2 to the trispanning orphan receptor of *Schistosoma haematobium* (Sh-TOR). Ecto-enzymes on the tegument of intravascular stages including the **** schistosomula and **** adults cleave extracellular ATPs that otherwise serve as damage-associated molecular patterns (DAMPs) as well as ADPs and inorganic polyphosphates (polyPs), thereby interfering with host pro-inflammatory and prothrombotic purinergic signaling. Eggs from the schistosomes *in copula* **** express proteases that may aid in egg egress in addition to Th2 immune responses. Granuloma formation with schistosome-induced fibrogenesis tends to limit tissue destruction brought about by egg deposition. However, disease severity is largely determined by Th17/Treg balance mediated by transforming growth factor-β (TGF-β).

Schistosomiasis is a neglected tropical disease whose transmission has been reported in 78 countries. This parasitic disease has been a public health problem as early as 5,000 years ago upon being discovered in Egyptian mummies (156). This long relationship between humans and the schistosome parasites has enabled the latter to adopt various strategies to successfully survive inside the host. Figure 1 shows the summary of proposed immunomodulatory armamentarium that schistosome parasites utilize in order to evade the host's immune responses, and thus facilitate infection. Understanding the mechanisms behind these immunomodulatory strategies will not only shed light on host-parasite interactions but also be useful in the development of novel treatments against the schistosome parasite.

Parasitic helminths like *Schistosoma* spp. are said to be capable of limiting intraspecific competition inside the host via concomitant immunity (157). Concomitant immunity is the production of effective anti-larval immunity that does not harm the existing adults. The adult worms might be “vaccinating” the host with cross-reactive antigens creating a barrier against new infection. This has been proven with an experimental study involving monkeys infected with adult schistosomes via surgical transplants (158). The monkeys showed resistance to cercarial challenge even though they were not exposed to any larval schistosome stages. However, another study has looked into potential mechanisms causing elimination of lung schistosomula in mice previously vaccinated with irradiated cercariae (159). Results show that the deflection of the parasites in the alveoli during migration was the reason many failed to mature in both vaccinated and unvaccinated mice, as no inflammatory reactions against the parasites have been found in the skin and lungs of the vaccinated mice (159). A better understanding of the role of immunomodulation in the early stage of schistosome infection might be the key in the production of a “true” effective anti-larval immunity against *Schistosoma*.

The 2-fold ability of the helminth worms to downregulate pro-inflammatory cytokines and skew Th1 to Th2 type immune responses has suggested their possible use in treating other illnesses such as autoimmune and inflammatory diseases, thus supporting the hygiene hypothesis (160). This hypothesis states that persons who never contract infections run the risk of developing autoimmune diseases, as infections facilitate the development and regulation of the immune system (161). Therefore, immunomodulatory molecules elaborated in response to the schistosome parasite can serve as potential tools to control overt immune responses.

Experimental studies have demonstrated the immunomodulatory effects of schistosome infection on arthritis (162–164), type 1 diabetes (165–168), Graves' disease (169), and airway allergies (170, 171). The therapeutic potentials of immunomodulatory molecules such as smCKBP might be used as selective manipulators of the immune system to prevent immune-mediated diseases (115). The schistosome-derived carbohydrate LNFPIII might be useful in treating type 2 diabetes as its administration in mice has improved glucose tolerance and insulin sensitivity (172), and in psoriasis as it induces Th2 immune response, and subsequent amelioration of skin lesions (173). Purified cystatin from *S. japonicum* has been shown to reduce inflammatory parameters and decrease the severity of trinitrobenzene sulfonic acid (TNBS)-induced colitis in mice, thereby demonstrating its potential therapeutic use in inflammatory bowel diseases (174). Taking advantage of these adaptive mechanisms of the schistosome parasite thus offers promise in the management of various autoimmune and inflammatory conditions. More immunomodulatory molecules and their interactomes and mechanisms need to be identified and characterized to develop effective drugs to achieve this end.

## Author Contributions

JA, VM, and PR contributed on the conception of the review paper. JA wrote the first draft of the manuscript. JA, VM, and PR wrote sections of the manuscript. All authors contributed to manuscript revision, read, and approved the submitted version.

## Conflict of Interest

The authors declare that the research was conducted in the absence of any commercial or financial relationships that could be construed as a potential conflict of interest.
